# Partial nephrectomy using porcine small intestinal submucosa

**DOI:** 10.1186/1477-7819-9-126

**Published:** 2011-10-12

**Authors:** Thomas J Schnoeller, Robert de Petriconi, Robert Hefty, Florian Jentzmik, Sandra Waalkes, Friedemann Zengerling, Mark Schrader, Andres J Schrader

**Affiliations:** 1 Department of Urology, Ulm University Medical Center, Prittwitzstrasse 43, D-89075 Ulm, Germany; 2 Department of Urology, Hannover Medical School, Carl-Neuberg-Str. 1, D-30625 Hannover, Germany

**Keywords:** renal cell carcinoma, nephron-sparing surgery, urinoma, postoperative hemorrhage, complications

## Abstract

**Background:**

Whenever technically feasible and oncologically justified, nephron-sparing surgery is the current standard of care for localized renal cell carcinomas (RCC). The main complications of partial nephrectomy, especially for large and centrally located tumors, are urinary leakage and parenchymal bleeding. We prospectively evaluated the pros and cons of using porcine small intestinal submucosa (SIS, Surgisis^®^) to close the renal defect after nephron-sparing surgery.

**Methods:**

We used Surgisis^® ^(Cook medical, Bloomington, IN, USA) to secure and compress the capsular defect after tumor resection in 123 patients submitted to 129 partial nephrectomies between August 2003 and February 2011.

**Results:**

The median tumor size was 3.7 cm (range 1.1-13.0 cm). Procedures were performed with cold ischemia in 24 cases (18.2%), with warm ischemia in 46 (35.6%), and without ischemia in 59 cases (44.8%). In the total group of patients, 4 (3.1%) developed urinary fistula, and only 2 (1.6%) required postoperative transfusions due to hemorrhage after the application of the small intestinal submucosa membrane.

**Conclusion:**

Small intestinal submucosa is an easy-to-use biomaterial for preventing complications such as postoperative bleeding and urinary fistula in nephron-sparing surgery, especially in cases where tumor excision causes significant renal capsular and/or renal pelvic defects.

## Background

Whenever technically feasible and oncologically justified, nephron-sparing surgery is the current standard of care for localized renal tumors of any size [1]. Its high status is based on substantial evidence from numerous studies confirming that postoperative morbidity such as cardiovascular disease, diabetes and renal failure can thus be avoided without significantly increasing the risk of tumor recurrence [2-5]. Nevertheless, the proportion of nephron-sparing interventions in most urological centers is still well below 50% [6]. One reason for this discrepancy may be that partial nephrectomy is sometimes technically demanding and occasionally involves significant complications in a percentage of patients that merits attention. The most prevalent adverse outcomes after partial nephrectomy include urinary leakage/fistula (1.3 - 9.1%) and parenchymal bleeding (0 - 7.9%) with higher complication rates after excision of larger tumors [7-15].

Porcine small intestinal submucosa (SIS, Surgisis^®^) is a natural acellular collagen-based biomaterial. The Surgisis^® ^membrane is increasingly used for different purposes in abdominal surgery [16,17], gynecology and obstetrics [18], and, more recently, urology [19-21].

We describe the use of this small intestinal submucosa membrane to optimize and facilitate closure of the collecting system and particularly the parenchymal defect and to minimize parenchymal bleeding after partial nephrectomy in a series of 123 patients.

## Materials and methods

**Figure 1:**
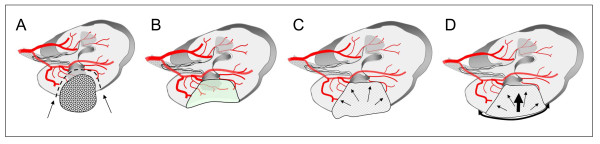
**Schematic illustration of a partial nephrectomy and subsequent application of Surgisis^® ^to optimize and facilitate the closure of the renal defect: **A) Tumor in place, B) kidney after tumor excision, C) creation of a phantom or placeholder volume using a hemostyptic agent, D) optimum hemostasis after fixation of Surgisis^® ^to apply homogeneous pressure onto the resection ground.

Surgisis^® ^(Cook medical, Bloomington, IN, USA) was used to close and secure the capsular defect in 123 patients who underwent 129 partial nephrectomies from August 2003 to March 2011 (Figure 1).

**Figure 2:**
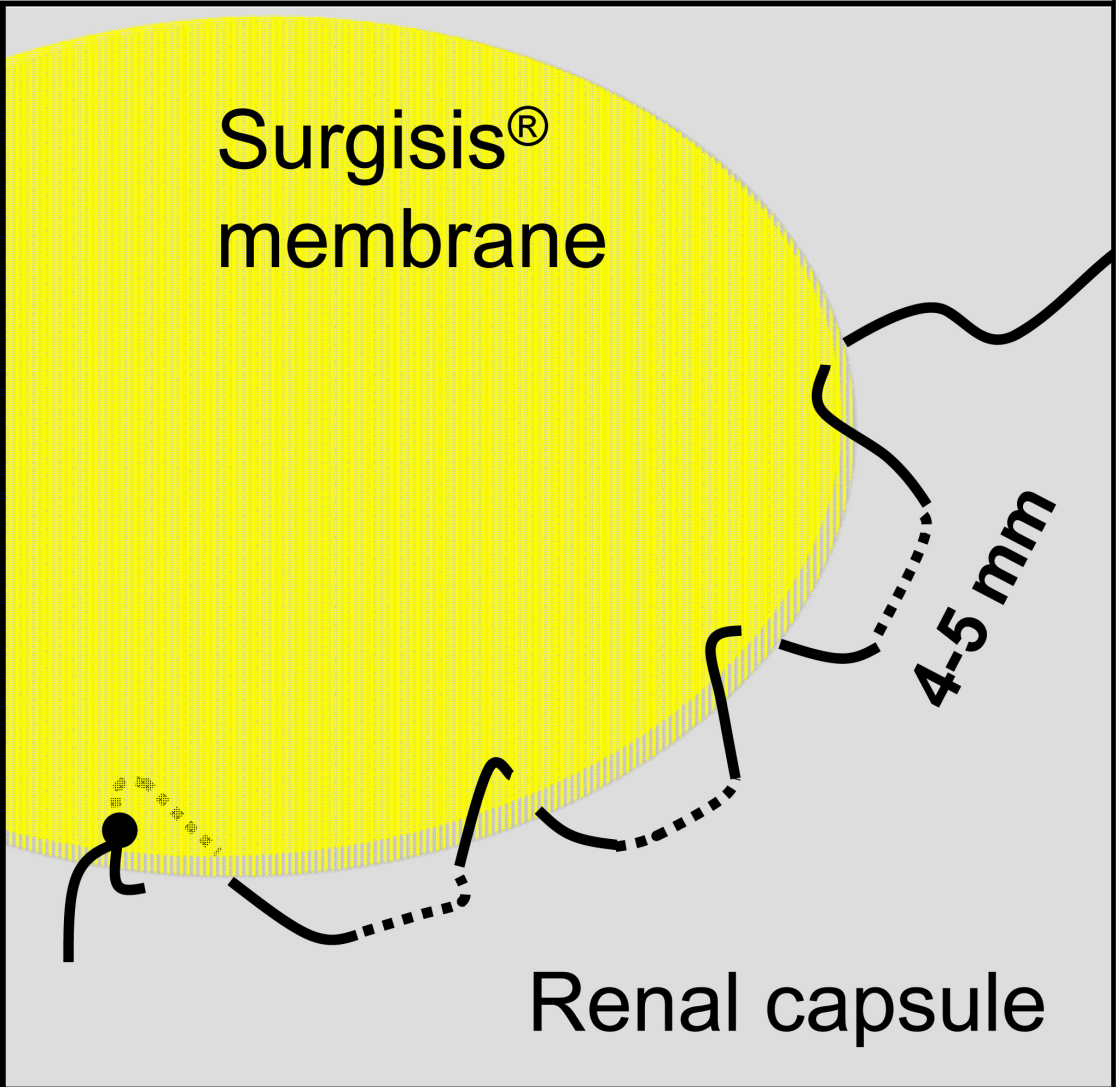
**Schematic illustration of the running suture technique mimicking the fixation of a drumhead to fix the Surgisis^® ^membrane to the renal capsule over the renal defect**.

After excising the tumor with a normal tissue margin using a Leriche dissector for blunt dissection as described earlier [20], we ligated or clipped visible blood vessels within the renal defect. The collecting system, if necessary, was closed with monofilament sutures. A pyelostoma or mono-J catheter was inserted in cases with large collecting system defects. The renal defect was then treated with a hemostyptic agent such as TachoSil^® ^(Nycomed, Unterscheissheim, Germany), FloSeal^® ^(Baxter, Deerfield, IL, USA), and/or Tabotamp^® ^(Johnson & Johnson Medical GmbH, Norderstedt, Germany). The hydrated Surgisis^® ^membrane was then cut into shape and tightly fixed over the parenchymal defect with a 3-0 Vicryl a running suture mimicking the fixation of a drumhead (Figure 2). Suturing of the renal surface was kept superficial, since deep stitches cause additional parenchymal scarring. If FloSeal^® ^was used for hemostasis, it was injected into the parenchymal defect beneath the Surgisis^® ^tissue graft just before finishing the running sutures securing the membrane. A percutaneous drain was placed to monitor postoperative bleeding and urinary leakage.

## Results

Depending on the tumor size and/or location within the kidney, procedures were performed with cold ischemia in 24/129 cases (18.6%), with warm ischemia in 46/129 (35.6%), and without ischemia in 59/129 (45.8%) cases. The median tumor size was 3.7 cm (range 1.1-13.0 cm). Thirty-one lesions were benign, and 98 were classified as RCC. In the latter group, staging revealed 94 pT1 tumors, 1 pT2 tumor, 2 pT3 tumors, and 1 pT4 tumor. It generally took about 5-10 min to fix the Surgisis^® ^membrane over the renal defect.

Four patients (3.1%) developed postoperative urinary fistulas, three of whom had tumors > 4 cm. However, the insertion of Mono-J and/or Double-J catheters achieved fistula closure without further intervention. Two patients (1.6%) with significant postoperative bleeding required reoperation or coiling of a segmental artery. The reason for reoperation was a bleeding venotomy suture line following *in situ *cold perfusion immediately after the primary surgical procedure.

**Figure 3:**
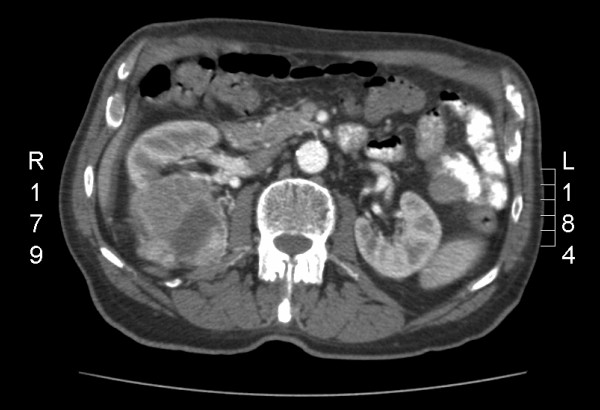
**Large clear cell renal cell carcinoma prior to nephron-sparing surgery in a patient with chronic renal failure**.

**Figure 4:**
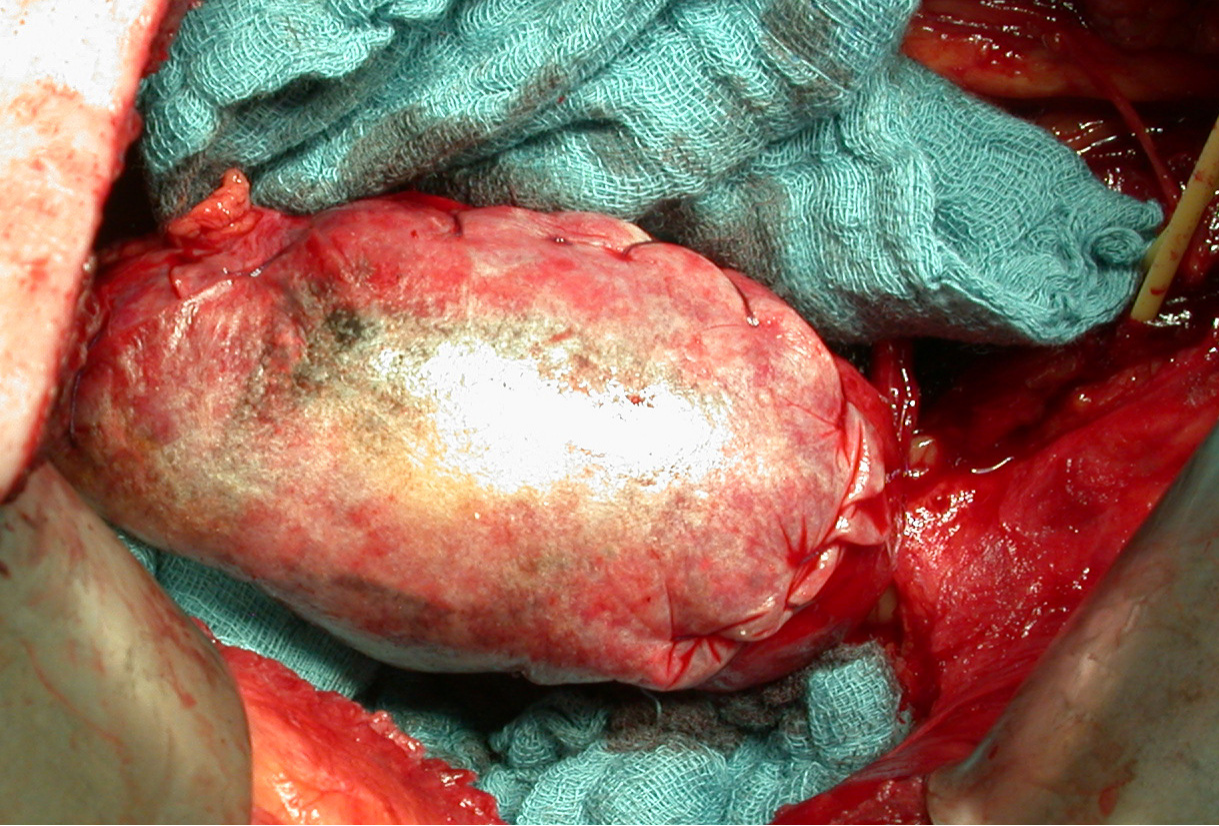
**Hydrated porcine small intestinal submucosa (Surgisis^®^) trimmed and tacked in place over the large renal parenchymal defect (cf. Figure 3), fixed with two running sutures**.

Figures 3 and 4 show pictures of a patient with chronic renal failure who suffered from a large renal cell carcinoma and was treated by partial nephrectomy. The renal defect was easily closed and controlled using a Surgisis^® ^membrane.

## Discussion

The incidence of localized renal cell carcinoma (RCC) continues to increase; these tumors account for up to 81% of all RCC at diagnosis, at least in urban areas [6]. Nephron-sparing surgery is now the recommended standard of care for localized resectable RCC of any size [1,22,23], although this is not yet reflected in routine clinical practice. A recently published population-based case-control study conducted by Miller et al. [6] in metropolitan Detroit and Chicago showed that until 2007 almost 80% of patients with localized RCC were treated by radical and only 20% by partial nephrectomy. This is alarming, since several trials have clearly demonstrated that total removal of the kidney was associated with a higher risk of postoperative renal failure [2], cardiovascular disease [3], diabetes [4] and even early death [3,5].

While it is generally agreed that a nephron-sparing approach should be adopted for surgical treatment of localized RCC, the optimal technique (open, laparoscopic, or robot-assisted) is still under debate and probably of secondary importance [10,24,25].

Despite the steadily decreasing incidence of postoperative adverse events after partial nephrectomy, mostly due to the development of novel hemostyptic agents, a significant number of complications still occur. In a 2007 study on partial nephrectomy in 1,800 patients (median tumor size 3 cm), Gill et al. [10] reported that 5-9% had postoperative urological complications, including hemorrhage (2-4%), urine leakage (2-3%), and renal failure (1-2%). Subsequent invasive procedures were required in 4-7% of all patients. Moreover, the complication rate seems to increase with the size of the defect after tumor excision [7].

Here we describe an improved technique for parenchymal defect closure in which the use of a modern hemostyptic agent is combined with Surgisis^® ^membrane placement to secure and compress the defect without causing significant additional renal parenchymal injury or scarring. In contrast to conventional surgical techniques for hemostasis, the use of a Surgisis^® ^membrane does not compromise blood flow in the parenchymal resection margin, since no parenchymal tissue has to be gathered or folded. The running suture technique mimicking the fixation of a drumhead (Figure 2) additionally enables maximal stability and prevents tearing of the suture from the renal capsule [26]. Another great advantage of this technique is the establishment of a phantom or placeholder volume beneath the membrane (Figure 1) which enables the establishment of homogenous pressure on the resection area. After > 120 application, we feel that this can most easily be achieved using Flowseal because of its swell volume of approximately 20% [27], which is achieved within only a few minutes and leads to an additional physical restriction of blood flow.

Significant postoperative bleeding from the tumor base occurred in 0.8% and urinary fistulas in 3.1% of our patient population, only. Thus the procedure described here may further reduce the complication rate of partial nephrectomy and helps to minimize the loss of functional renal tissue. We also feel that the use of this simplified and safe surgical technique could further increase the proportion of patients undergoing nephron-sparing surgery, which has surely become one of the most important goals in the treatment of localized RCC.

Also noteworthy in this context is a recent study by O'Connor et al. [21] who reported that one of their 24 patients submitted to partial nephrectomy using a Surgisis^® ^membrane had to undergo a second partial nephrectomy for a new ipsilateral tumor 9 months after the original procedure. Intraoperatively, the reoperated kidney appeared absolutely normal with total incorporation of Surgisis^® ^into the collecting system and parenchyma. As in animal models, this membrane appeared to have been replaced by native tissue with no significant local fibrosis [28]. This may prove beneficial, particularly in cases of angiomyolipoma or familial renal tumors that may require multiple, ipsilateral partial nephrectomies [21].

A disadvantage of the surgical technique described here is the expense involved; we pay approximately € 204 for one Surgisis^® ^membrane. In addition, the suitability of this technique for endoscopic tumor enucleations still has to be assessed.

## Conclusion

Surgisis^® ^membrane placement combined with the use of a conventional hemostyptic agent is effective, free from side effects, and very easy to perform; it is indicated especially for safe closure of large renal capsular defects. Therefore, currently a clinical trial is being planned in cooperation with the German Renal Cell Cancer Network, in which the application of Surgisis^® ^will be evaluated vs. standard closure of renal defects for tumors with a diameter > 4 cm. We do hope that the establishment of novel techniques such as the application of Surgisis^® ^will further increase the proportion of patients undergoing nephron-sparing surgery.

## Competing interests

The authors declare that they have no competing interests.

## Authors' contributions

TJM provided the idea, collected the data, and contributed by writing parts of the manuscript; RP performed most operations and supplied Figures 1+2; RH and FJ performed several partial nephrectomies using surgisis^® ^and helped to discuss the data; SW, FZ, and MS contributed mainly in designing, literature review, and writing of the manuscript; AJS, the corresponding author of this study, planed, corrected, and approved the written work, and supplied Figures 3+4. All authors read and approved the final manuscript.
